# Author Correction: Nasal airway transcriptome-wide association study of asthma reveals genetically driven mucus pathobiology

**DOI:** 10.1038/s41467-022-33097-z

**Published:** 2022-10-03

**Authors:** Satria P. Sajuthi, Jamie L. Everman, Nathan D. Jackson, Benjamin Saef, Cydney L. Rios, Camille M. Moore, Angel C. Y. Mak, Celeste Eng, Ana Fairbanks–Mahnke, Sandra Salazar, Jennifer Elhawary, Scott Huntsman, Vivian Medina, Deborah A. Nickerson, Soren Germer, Michael C. Zody, Gonçalo Abecasis, Hyun Min Kang, Kenneth M. Rice, Rajesh Kumar, Noah A. Zaitlen, Sam Oh, NHLBI Trans–Omics for Precision Medicine (TOPMed) Consortium, José Rodríguez–Santana, Esteban G. Burchard, Max A. Seibold

**Affiliations:** 1 Center for Genes, Environment, and Health, National Jewish Health, Denver, CO USA; 2 Department of Biomedical Research, National Jewish Health, Denver, CO USA; 3 Department of Biostatistics and Informatics, University of Colorado, Denver, CO USA; 4 Department of Medicine, University of California–San Francisco, San Francisco, CA USA; 5 Centro de Neumología Pediátrica, San Juan, PR USA; 6 Department of Genome Sciences, University of Washington, Seattle, WA USA; 7 New York Genome Center, New York, NY USA; 8 Center for Statistical Genetics, University of Michigan, Ann Arbor, MI USA; 9 Department of Biostatistics, University of Washington, Seattle, WA USA; 10 Ann and Robert H. Lurie Children’s Hospital of Chicago, Department of Pediatrics, Northwestern University, Chicago, IL USA; 11 Department of Neurology and Computational Medicine, University of California Los Angeles, Los Angeles, CA USA; 12 Department of Bioengineering and Therapeutic Sciences, University of California–San Francisco, San Francisco, CA USA; 13 Department of Pediatrics, National Jewish Health, Denver, CO USA; 14 Division of Pulmonary Sciences and Critical Care Medicine, University of Colorado School of Medicine, Aurora, CO USA

**Keywords:** Gene regulation, Gene expression, Asthma

Correction to: *Nature Communications*10.1038/s41467-022-28973-7,published online 28 March 2022

The original version of this article used a script to annotate the genotype matrix with dbSNP Reference SNP numbers (rs ids), that contained an error that resulted in 130,911 variants within a 26MB region of chromosome 22 not being annotated with an rs id. Additionally, 452,551 variants that overlapped with an indel variant received the wrong rs ids and 409,126 variants that overlapped with an indel variant did not get annotated with an rs id. In the corrected version, the script has been fixed and the analyses have been redone. The following have been corrected in both the PDF and HTML versions of the Article:

In the original version of the article, the second sentence of the abstract incorrectly stated “Our airway analysis identified 95 asthma genes”. The correct version replaces “95” with “102”.

In the original version of the article, in the “Results” subsection “Nasal epithelium TWAS identifies COA/AOA genes”, the original first paragraph incorrectly stated “We identified 88 significant TWAS genes (Bonferroni-corrected *p*–value threshold = 3.99e−6) for COA and 18 genes for AOA, for a total of 95 unique genes (11 shared between COA and AOA, Fig. 2a, Table 1)”, which has been replaced with “We identified 93 significant TWAS genes (Bonferroni-corrected *p*-value threshold = 3.99e−6) for COA and 21 genes for AOA, for a total of 102 unique genes (12 shared between COA and AOA, Fig. 2a, Table 1)”. The original version of this paragraph also incorrectly stated “We identified at least one nasal TWAS gene in close proximity (1 Mb) to 32 of the 89 independent COA risk loci (36%) and to 13 of the 40 independent AOA risk loci (33%, Supplementary Data 3). We also identified 8 COA TWAS genes (SYT13, IQGAP1, FOXA3, TIPARP, LOC115110, CCDC66, AAMDC, RFTN2) and a single AOA TWAS gene (C7ORF26) that were not within a Mb of any GWAS risk variant (Fig. 2a)”; which has been replaced with “We identified at least one nasal TWAS gene in close proximity (1 Mb) to 33 of the 89 independent COA risk loci (37%) and to 13 of the 40 independent AOA risk loci (33%, Supplementary Data 3). We also identified 9 COA TWAS genes (SYT13, IQGAP1, FOXA3, TIPARP, LOC115110, CCDC66, AAMDC, RFTN2, IL7R) and 3 AOA (ZDHHC18, C7ORF26, LOC100130476) TWAS genes that were not within a Mb of any GWAS risk variant (Fig. 2a)”.

The second paragraph of the “Results” subsection “Nasal epithelium TWAS identifies COA/AOA genes” incorrectly stated “Comparing overlap in nasal TWAS genes with the other TWAS tissues, we found that 51 of the 88 COA and 11 of the 18 AOA nasal TWAS genes were only identified in that tissue (Fig. 2b, c)”; which has been replaced with “Comparing overlap in nasal TWAS genes with the other TWAS tissues, we found that 52 of the 93 COA and 13 of the 21 AOA nasal TWAS genes were only identified in that tissue (Figure 2b,c)”.

The third paragraph of the “Results” subsection “Nasal epithelium TWAS identifies COA/AOA genes” incorrectly stated “One of the nasal-specific TWAS genes, interleukin-33 (IL33), for which increased expression was associated with increased asthma risk, was the most strongly associated AOA TWAS gene and the third most strongly associated TWAS gene for COA”. The correct version replaces “third” with “second”.

The fourth paragraph the “Results” subsection “Nasal epithelium TWAS identifies COA/AOA genes” incorrectly stated “Our nasal TWAS analysis identified 14 COA-associated genes at the 17q21 locus, including 7 that were observed only in the nasal TWAS (LINC00672, RPL19, MED1, GRB7, IKZF3, PSMD3, RARA-AS1)”. The strongest nasal TWAS gene association with COA was for GSDMB (*p* = 9.1e−124), which also showed strong colocalization with the GWAS signal (PP4 = 0.99). Interestingly, GSDMB expression was associated in COA TWAS analyses for all GTEx tissues tested. In contrast, the IKZF3 gene, which exhibited the second strongest nasal COA association (*p* = 3.9e−106), was only detected in the nasal analysis (Figure 3a). In the correct version, this is replaced with “Our nasal TWAS analysis identified 12 COA-associated genes at the 17q21 locus, including 6 that were observed only in the nasal TWAS (LINC00672, MED1, GRB7, IKZF3, PSMD3, RARA-AS1). The strongest nasal TWAS gene association with COA was for GSDMB (*p* = 5.5e−124), which also showed strong colocalization with the GWAS signal (PP4 = 0.99). Interestingly, GSDMB expression was associated in COA TWAS analyses for all GTEx tissues tested. In contrast, the IKZF3 gene, which exhibited the third strongest nasal COA association (*p* = 1.7e−74), was only detected in the nasal analysis (Figure 3a)”.

In the “Results” subsection “A MUC5AC asthma risk eQTL exerts trans-effects on mucus secretory cell machinery”, the first paragraph originally stated “Intriguingly, we found that two of the TWAS genes that were unique to the nasal airway AOA analysis encode MUC2 (*p* = 2.5 x 10–7) and MUC5AC (*p* = 4.7 x 10–13)”. The correct version replaces “(*p* = 2.5 x 10–7)” with “(*p* = 1.7 x 10–7)” and “(*p* = 4.7 x 10−13)” with “(*p* = 5.1 x 10–13)”.

The fourth paragraph of the “Results” subsection “A MUC5AC asthma risk eQTL exerts trans–effects on mucus secretory cell machinery” incorrectly stated “we found that MUC5AC was remarkably either one among only five (for phlegm) or the only (for cough) significantly associated gene, with the same rs12788104 marker LD block being positively associated with report of the trait (Supplementary Data 9)”. The correct version replaces “five” with “seven”.

In the “Results” subsection “A FOXA3 asthma risk eQTL drives metaplastic mucus secretory expression”, the first paragraph incorrectly stated “Also among the nasal-specific TWAS genes (COA *p* = 3.1e−7, AOA *p* = 2.1e−5) was FOXA3, a transcription factor whose expression is known to be induced by T2 inflammation”. The correct version replaces “2.1e−5” with “2.2e−5”.

The first paragraph of the “Discussion” incorrectly stated “Here, we have performed the first TWAS analysis for asthma using the nasal airway epithelium, resulting in identification of 108 COA and AOA risk genes”. The correct version replaces “108” with “114”. That paragraph also incorrectly stated “Moreover, our airway TWAS identified at least one significant gene for 32 of the 89 COA risk loci (36%)”. The correct version replaces “32” with “33” and “36” with “37”.

The second paragraph of that section incorrectly stated “This analysis only identified 55 asthma TWAS genes; with only 1 of the 11 AOA and 3 of 51 COA nasal-specific TWAS genes we identified among them”. The correct version replaces this sentence with “This analysis only identified 55 asthma TWAS genes; with only 1 of the 13 AOA and 5 of 52 COA nasal-specific TWAS genes identified among them”.

The third paragraph of that section incorrectly stated “our results suggest additional involvement of the locus based on our identification of 15 COA-associated genes across the six tissue types examined, including 14 from the airway, of which seven were airway-specific”. The correct version now states “our results suggest additional involvement of the locus based on our identification of 24 COA-associated genes across the six tissue types examined, including 12 from the airway, of which six were airway-specific”.

The seventh paragraph of that section incorrectly stated “In addition to FOXA3, we identified seven other COA and one other AOA genes”. The correct version now states “In addition to FOXA3, we identified eight other COA and three other AOA genes”.

In the “Methods” sub-section “Cis-eQTL Analysis”, the original version of the manuscript incorrectly omitted the statement “Variants were annotated using DBSNP 150”. The correct version now adds this statement.

The original version of the article contained errors in Fig. 2. The correct version of Fig. 2 is:

**Figure:**
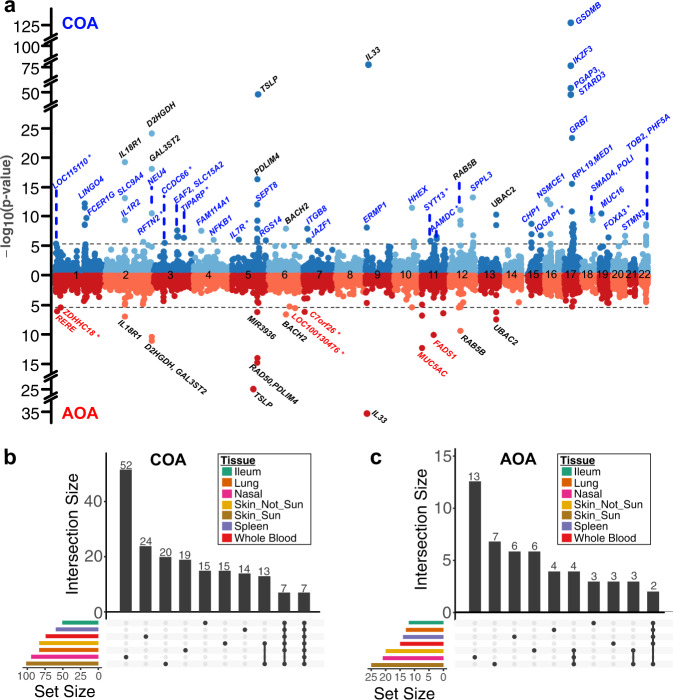

which replaces the previous incorrect version:

**Figure:**
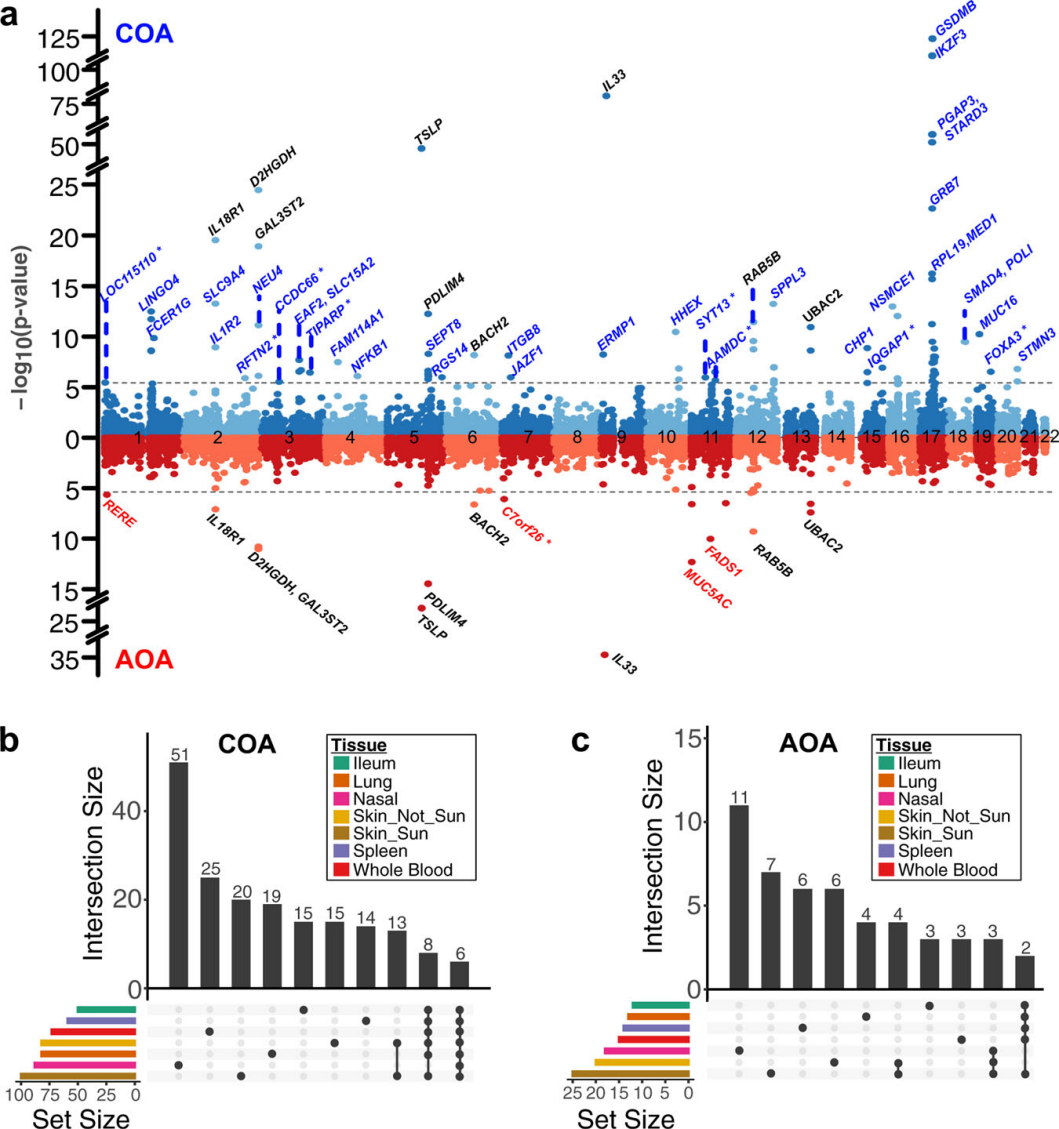

The original version of the article contained errors in Fig. 3. The correct version of Fig. 3 is:

**Figure:**
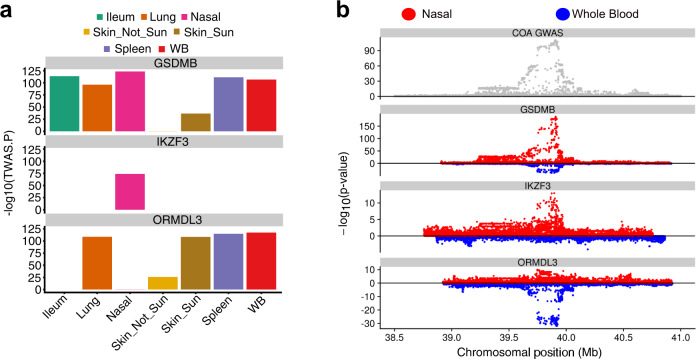

which replaces the previous incorrect version:

**Figure:**
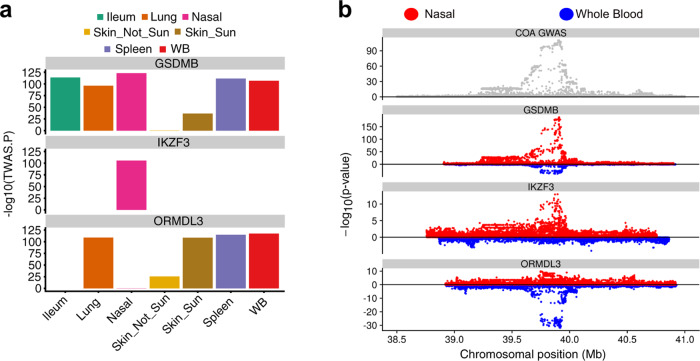

The original version of the article contained errors in Table 1. The correct version of Table 1 is:

**Table:** 

				Childhood Onset Asthma				Adult Onset Asthma				Nasal Specific?
No.	Gene	Position (Hg38)	HSQ	TWAS.Z	TWAS.P	PP3	PP4	TWAS.Z	TWAS.P	PP3	PP4	
**Childhood Onset Asthma**												
1	LOC115110	1:2549920–2557011	0.46	4.63	3.73E−06	0.02	0.98	3.15	1.65E−03	NA	NA	Y
2	RIIAD1	1:151721537–151729606	0.54	−5.99	2.09E−09	1.00	0.00	−1.87	6.12E−02	NA	NA	Y
3	MRPL9	1:151759643–151763916	0.14	−5.96	2.45E−09	1.00	0.00	−1.70	8.82E−02	NA	NA	Y
4	LINGO4	1:151800265–151805442	0.42	7.21	5.39E−13	1.00	0.00	0.88	3.77E−01	NA	NA	N
5	FLG-AS1	1:152313459–152366692	0.31	−7.00	2.60E−12	1.00	0.00	−1.52	1.29E−01	NA	NA	N
6	FCER1G	1:161215297–161219248	0.19	6.40	1.57E−10	0.00	1.00	3.70	2.12E−04	NA	NA	N
7	IL1R2	2:101991844–102028422	0.07	−6.27	3.57E−10	0.99	0.00	−3.81	1.41E−04	NA	NA	Y
8	SLC9A4	2:102473303–102533972	0.21	−7.50	6.55E−14	1.00	0.00	−4.38	1.18E−05	NA	NA	Y
9	RFTN2	2:197570803–197675860	0.31	−4.77	1.86E−06	0.07	0.93	−4.06	4.87E−05	NA	NA	N
10	NEU4	2:241809065–241817413	0.49	6.67	2.51E−11	1.00	0.00	2.92	3.55E−03	NA	NA	Y
11	CCDC66	3:56557156–56621820	0.50	−4.69	2.75E−06	0.08	0.92	−3.12	1.82E−03	NA	NA	Y
12	EAF2	3:121835187–121886526	0.10	−5.16	2.48E−07	0.50	0.50	−2.73	6.41E−03	NA	NA	N
13	SLC15A2	3:121894324–121944187	0.46	5.61	2.02E−08	0.15	0.85	2.89	3.80E−03	NA	NA	Y
14	RUVBL1	3:128080957–128123828	0.29	−5.18	2.22E−07	0.97	0.03	−2.28	2.26E−02	NA	NA	Y
15	TIPARP	3:156674416–156706768	0.08	5.08	3.72E−07	0.15	0.84	2.56	1.05E−02	NA	NA	Y
16	FAM114A1	4:38867733–38945744	0.13	−5.58	2.34E−08	0.37	0.06	1.39	1.64E−01	NA	NA	N
17	NFKB1	4:102501329–102617302	0.40	−4.93	8.35E−07	0.21	0.79	−1.37	1.71E−01	NA	NA	Y
18	IL7R	5:35856875–35879603	0.07	−4.95	7.49E−07	0.17	0.69	−2.81	4.99E−03	NA	NA	Y
19	LOC553103	5:132311276–132369916	0.43	−4.76	1.91E−06	1.00	0.00	−3.61	3.12E−04	NA	NA	Y
20	SLC22A5	5:132369709–132395614	0.37	5.03	4.86E−07	1.00	0.00	3.16	1.60E−03	NA	NA	N
21	SEPT8	5:132750817–132777869	0.12	6.24	4.43E−10	1.00	0.00	4.11	3.90E−05	NA	NA	N
22	RGS14	5:177357843–177372598	0.50	−4.89	1.02E−06	0.26	0.73	−2.96	3.05E−03	NA	NA	N
23	ITGB8	7:20331102–20415759	0.08	−5.73	1.02E−08	0.64	0.29	−3.81	1.40E−04	NA	NA	Y
24	JAZF1	7:27830574–28180818	0.19	4.89	1.02E−06	1.00	0.00	2.92	3.45E−03	NA	NA	Y
25	ERMP1	9:5784572–5833081	0.38	5.80	6.67E−09	1.00	0.00	4.23	2.35E−05	NA	NA	Y
26	HHEX	10:92689924–92695651	0.25	6.98	2.90E−12	0.02	0.98	4.60	4.19E−06	NA	NA	Y
27	WBP1L	10:102743970–102816264	0.40	4.80	1.55E−06	1.00	0.00	0.96	3.37E−01	NA	NA	Y
28	SYT13	11:45240302–45286333	0.45	−4.86	1.15E−06	0.03	0.97	−2.88	3.99E−03	NA	NA	Y
29	MYO7A	11:77128264–77215241	0.12	5.18	2.21E−07	0.99	0.00	3.08	2.09E−03	NA	NA	Y
30	AAMDC	11:77821162–77872352	0.05	−5.00	5.83E−07	0.26	0.56	−1.06	2.87E−01	NA	NA	Y
31	RPS26	12:56041902–56044223	0.33	6.87	6.58E−12	1.00	0.00	4.59	4.54E−06	NA	NA	N
32	NABP2	12:56224341–56229854	0.07	−5.99	2.06E−09	0.63	0.03	−4.45	8.69E−06	NA	NA	Y
33	SPPL3	12:120762510–120904352	0.19	7.54	4.86E−14	0.97	0.03	2.79	5.27E−03	NA	NA	N
34	ABCB9	12:122920951–122966509	0.14	−4.74	2.18E−06	0.96	0.05	−0.76	4.45E−01	NA	NA	N
35	CDK2AP1	12:123260970–123272316	0.44	5.26	1.41E−07	0.48	0.52	0.86	3.87E−01	NA	NA	N
36	BAHD1	15:40439721–40468242	0.05	4.74	2.19E−06	0.62	0.08	0.83	4.07E−01	NA	NA	Y
37	CHP1	15:41231239–41281885	0.33	−6.04	1.53E−09	0.79	0.21	−2.73	6.26E−03	NA	NA	N
38	OIP5-AS1	15:41284003–41299597	0.19	−4.69	2.70E−06	0.87	0.13	−1.35	1.78E−01	NA	NA	N
39	ITPKA	15:41493858–41503559	0.21	5.36	8.54E−08	0.01	0.99	2.83	4.72E−03	NA	NA	Y
40	IQGAP1	15:90388241–90502243	0.41	−5.27	1.34E−07	0.01	0.99	−1.10	2.72E−01	NA	NA	N
41	DEXI	16:10928891–10942400	0.32	−7.41	1.23E−13	1.00	0.00	−2.91	3.66E−03	NA	NA	Y
42	NSMCE1	16:27224994–27268792	0.26	7.19	6.65E−13	1.00	0.00	3.11	1.84E−03	NA	NA	Y
43	IL4R	16:27313909–27364778	0.17	5.36	8.17E−08	0.66	0.33	2.59	9.49E−03	NA	NA	N
44	ATP2A1	16:28878488–28904509	0.08	−4.62	3.80E−06	0.30	0.66	–0.97	3.33E−01	NA	NA	Y
45	LINC00672	17:38925168–38929384	0.10	6.78	1.19E−11	0.63	0.01	−1.31	1.89E−01	NA	NA	Y
46	MED1	17:39404285–39451274	0.17	8.20	2.46E−16	1.00	0.00	−0.11	9.16E−01	NA	NA	Y
47	STARD3	17:39637080–39664201	0.35	14.75	3.30E–49	1.00	0.00	2.05	4.07E−02	NA	NA	N
48	PGAP3	17:39671122–39688070	0.61	15.28	9.73E–53	1.00	0.00	1.54	1.24E−01	NA	NA	N
49	ERBB2	17:39688084–39728662	0.32	13.38	7.45E–41	1.00	0.00	0.48	6.33E−01	NA	NA	N
50	GRB7	17:39737909–39747285	0.09	10.14	3.85E–24	0.99	0.00	−2.49	1.29E−02	NA	NA	Y
51	IKZF3	17:39757715–39864188	0.11	−18.26	1.70E–74	0.42	0.58	−4.54	5.66E−06	NA	NA	Y
52	GSDMB	17:39904595–39918650	0.52	23.68	5.47E−124	0.01	0.99	2.71	6.70E−03	NA	NA	N
53	GSDMA	17:39962973–39977766	0.34	−13.40	5.74E–41	1.00	0.00	−2.93	3.43E−03	NA	NA	N
54	PSMD3	17:39980768–39997960	0.06	11.33	9.54E–30	1.00	0.00	2.18	2.95E−02	NA	NA	Y
55	MED24	17:40019097–40054636	0.45	5.46	4.73E−08	1.00	0.00	0.02	9.85E−01	NA	NA	N
56	RARA-AS1	17:40340867–40343136	0.06	5.84	5.11E−09	0.98	0.01	1.63	1.04E−01	NA	NA	Y
57	STAT5A	17:42287547–42311942	0.13	−4.69	2.76E−06	0.72	0.28	−3.43	5.95E−04	NA	NA	Y
58	HEXIM1	17:45147317–45152101	0.27	−6.01	1.80E−09	1.00	0.00	−1.24	2.14E−01	NA	NA	Y
59	SPATA32	17:45254393–45262112	0.73	−5.21	1.88E−07	0.98	0.02	−2.71	6.67E−03	NA	NA	N
60	LRRC37A4P	17:45505883–45520523	0.32	5.66	1.55E−08	1.00	0.00	0.17	8.64E−01	NA	NA	N
61	LOC644172	17:45600103–45601862	0.10	−5.71	1.16E−08	1.00	0.00	−0.46	6.44E−01	NA	NA	Y
62	MGC57346	17:45620329–45637963	0.22	−5.52	3.34E−08	1.00	0.00	−0.39	6.97E−01	NA	NA	Y
63	CRHR1-IT1	17:45638975–45646229	0.26	−5.63	1.82E−08	1.00	0.00	−0.42	6.78E−01	NA	NA	N
64	MAPT	17:45894382–46028333	0.11	4.84	1.32E−06	1.00	0.00	−0.09	9.29E−01	NA	NA	N
65	KANSL1	17:46029916–46225374	0.14	−5.19	2.06E−07	1.00	0.00	−0.18	8.55E−01	NA	NA	N
66	KANSL1-AS1	17:46193573–46196723	0.29	−5.22	1.75E−07	1.00	0.00	−0.07	9.47E−01	NA	NA	N
67	LRRC37A	17:46295131–46337794	0.40	−6.01	1.85E−09	1.00	0.01	−0.16	8.72E−01	NA	NA	N
68	ZNF652	17:49289206–49362473	0.46	−5.72	1.07E−08	0.97	0.03	−3.31	9.29E−04	NA	NA	Y
69	SPOP	17:49598884–49678163	0.08	5.03	4.87E−07	0.60	0.02	4.09	4.25E−05	NA	NA	Y
70	SMAD4	18:51030213–51085041	0.25	−6.63	3.43E−11	0.06	0.94	−2.50	1.25E−02	NA	NA	N
71	POLI	18:54269479–54298234	0.52	6.28	3.49E−10	0.41	0.60	3.07	2.17E−03	NA	NA	N
72	MUC16	19:8848844–8981342	0.25	−6.66	2.69E−11	0.05	0.96	−2.77	5.53E−03	NA	NA	Y
73	FOXA3	19:45864260–45873797	0.42	5.12	3.13E−07	0.03	0.97	4.24	2.22E−05	NA	NA	Y
74	STMN3	20:63639705––63653610	0.23	−5.23	1.65E−07	0.03	0.97	−3.36	7.75E−04	NA	NA	N
75	LIME1	20:63735701–63739107	0.18	4.74	2.14E−06	0.72	0.28	3.00	2.66E−03	NA	NA	Y
76	RANGAP1	22:41244777–41286251	0.21	5.20	2.00E−07	0.58	0.42	2.59	9.63E−03	NA	NA	N
77	TOB2	22:41433488–41447023	0.05	−6.02	1.74E−09	0.05	0.95	−3.87	1.09E−04	NA	NA	Y
78	PHF5A	22:41459717–41468704	0.05	5.92	3.22E−09	0.27	0.73	4.15	3.34E−05	NA	NA	Y
79	XRCC6	22:41621163–41664048	0.04	−5.03	4.80E−07	0.34	0.59	−2.83	4.60E−03	NA	NA	N
80	C22orf46	22:41690543–41698136	0.13	−4.78	1.76E−06	0.84	0.16	−2.65	7.96E−03	NA	NA	Y
81	MEI1	22:41699514–41799455	0.16	4.66	3.20E−06	0.86	0.14	2.80	5.19E−03	NA	NA	N
**Shared**												
82	IL18R1	2:102356283–102398775	0.08	−9.16	5.05E–20	0.54	0.05	−5.31	1.11E−07	0.56	0.02	N
83	D2HGDH	2:241734579–241768816	0.64	10.31	6.63E–25	1.00	0.00	6.61	3.85E−11	1.00	0.00	N
84	GAL3ST2	2:241776825–241804287	0.43	8.87	7.02E−19	1.00	0.00	6.83	8.41E−12	1.00	0.00	Y
85	TSLP	5:111070080–111078024	0.34	14.68	8.88E–49	1.00	0.00	10.29	7.77E–25	1.00	0.00	N
86	PDLIM4	5:132257658–132273454	0.17	7.17	7.77E−13	1.00	0.00	7.97	1.64E−15	0.99	0.01	N
87	MIR3936	5:132365490–132365599	0.09	−5.74	9.74E−09	1.00	0.00	−4.98	6.52E−07	1.00	0.00	Y
88	RAD50	5:132556924–132644621	0.12	−8.41	4.00E−17	0.83	0.02	−7.74	9.91E−15	0.10	0.89	N
89	BACH2	6:89926528–90296908	0.09	5.73	1.01E−08	0.14	0.84	5.15	2.61E−07	0.14	0.84	Y
90	IL33	9:6215786–6257983	0.25	18.84	3.67E–79	0.00	1.00	12.41	2.18E–35	0.01	0.99	Y
91	RAB5B	12:55973913–55996683	0.05	6.23	4.57E−10	0.42	0.15	6.25	4.22E−10	0.07	0.86	Y
92	UBAC2-AS1	13:99196374–99200757	0.09	5.96	2.56E−09	0.14	0.86	5.51	3.62E−08	0.28	0.72	Y
93	UBAC2	13:99200425–99386499	0.29	−6.60	4.20E−11	0.04	0.96	−4.98	6.32E−07	0.20	0.80	Y
**Adult Onset Asthma**												
94	RERE	1:8352404–8817640	0.14	4.30	1.71E−05	NA	NA	4.91	8.90E−07	0.02	0.98	N
95	ZDHHC18	1:26826710–26855720	0.07	1.27	2.04E−01	NA	NA	4.62	3.85E−06	0.09	0.86	N
96	LOC100130476	6:137823670–137868233	0.08	2.60	9.38E−03	NA	NA	4.67	3.07E−06	0.29	0.70	Y
97	C7orf26	7:6590021–6608726	0.06	2.03	4.20E−02	NA	NA	4.96	7.05E−07	0.23	0.47	Y
98	MUC2	11:1074875–1110508	0.21	−2.10	3.56E−02	NA	NA	−5.24	1.65E−07	0.02	0.98	Y
99	MUC5AC	11:1157953–1201141	0.32	2.74	6.21E−03	NA	NA	7.22	5.13E−13	0.02	0.98	Y
100	FADS1	11:61799625–61817057	0.32	1.62	1.05E−01	NA	NA	6.49	8.49E−11	0.04	0.96	Y
101	SIK2	11:111602391–111726917	0.27	−2.63	8.44E−03	NA	NA	−5.06	4.10E−07	0.21	0.79	N
102	HDAC7	12:47782711–47819980	0.16	−0.98	3.29E−01	NA	NA	−4.62	3.92E−06	0.39	0.60	Y

which replaces the previous incorrect version:

**Table:** 

				Childhood Onset Asthma				Adult Onset Asthma				Nasal Specific?
No.	Gene	Position (Hg38)	HSQ	TWAS.Z	TWAS.P	PP3	PP4	TWAS.Z	TWAS.P	PP3	PP4	
**Childhood Onset Asthma**												
1	LOC115110	1:2549920–2557011	0.46	4.63	3.73E−06	0.02	0.98	3.15	1.65E−03	NA	NA	T
2	RIIAD1	1:151721537–151729606	0.54	−5.94	2.81E−09	1.00	0.00	−1.90	5.75E−02	NA	NA	T
3	MRPL9	1:151759643–151763916	0.14	−5.97	2.45E−09	1.00	0.00	−1.70	8.82E−02	NA	NA	T
4	LINGO4	1:151800265–151805442	0.42	7.28	3.39E−13	1.00	0.00	0.91	3.65E−01	NA	NA	F
5	FLG–AS1	1:152313459–152366692	0.31	−7.04	1.93E−12	1.00	0.00	−1.57	1.16E−01	NA	NA	F
6	FCER1G	1:161215297–161219248	0.19	6.41	1.42E−10	0.00	1.00	3.69	2.26E−04	NA	NA	F
7	IL1R2	2:101991844–102028422	0.07	−6.08	1.19E−09	0.99	0.00	−3.70	2.20E−04	NA	NA	T
8	SLC9A4	2:102473303–102533972	0.21	−7.52	5.64E−14	1.00	0.00	−4.43	9.53E−06	NA	NA	T
9	RFTN2	2:197570803–197675860	0.31	−4.83	1.33E−06	0.07	0.93	−4.11	3.90E−05	NA	NA	F
10	NEU4	2:241809065–241817413	0.49	6.84	7.69E−12	1.00	0.00	3.15	1.66E−03	NA	NA	T
11	LINC01237	2:241881363–242078722	0.05	−4.94	7.90E−07	0.99	0.00	−1.34	1.82E−01	NA	NA	T
12	CCDC66	3:56557156–56621820	0.50	−4.66	3.10E−06	0.08	0.92	−3.10	1.92E−03	NA	NA	T
13	EAF2	3:121835187–121886526	0.10	−5.13	2.97E−07	0.48	0.52	−2.72	6.52E−03	NA	NA	F
14	SLC15A2	3:121894324–121944187	0.46	5.61	2.08E−08	0.14	0.86	2.89	3.86E−03	NA	NA	T
15	RUVBL1	3:128080957–128123828	0.29	−5.18	2.17E−07	0.97	0.03	−2.33	2.00E−02	NA	NA	T
16	TIPARP	3:156674416–156706768	0.08	5.08	3.72E−07	0.14	0.84	2.56	1.05E−02	NA	NA	T
17	FAM114A1	4:38867733–38945744	0.13	−5.52	3.46E−08	0.37	0.06	1.48	1.38E−01	NA	NA	F
18	NFKB1	4:102501329–102617302	0.40	−4.93	8.35E−07	0.20	0.80	−1.37	1.71E−01	NA	NA	T
19	LOC553103	5:132311276–132369916	0.43	−4.79	1.68E−06	1.00	0.00	−3.70	2.15E−04	NA	NA	T
20	MIR3936	5:132365490–132365599	0.09	−5.16	2.49E−07	1.00	0.00	−4.30	1.73E−05	NA	NA	T
21	SLC22A5	5:132369709–132395614	0.37	5.01	5.33E−07	1.00	0.00	3.10	1.93E−03	NA	NA	F
22	SEPT8	5:132750817–132777869	0.12	5.84	5.30E−09	1.00	0.00	3.96	7.51E−05	NA	NA	F
23	UQCRQ	5:132866627–132868844	0.21	−4.92	8.69E−07	0.33	0.12	−3.35	7.94E−04	NA	NA	T
24	RGS14	5:177357843–177372598	0.50	−4.87	1.10E−06	0.26	0.73	−2.97	2.96E−03	NA	NA	F
25	ITGB8	7:20331102–20415759	0.08	−5.77	7.73E−09	0.64	0.29	−3.58	3.39E−04	NA	NA	T
26	JAZF1	7:27830574–28180818	0.19	4.87	1.12E−06	1.00	0.00	2.90	3.72E−03	NA	NA	T
27	ERMP1	9:5784572–5833081	0.38	5.81	6.17E−09	1.00	0.00	4.23	2.30E−05	NA	NA	T
28	HHEX	10:92689924–92695651	0.25	6.62	3.54E−11	0.02	0.98	4.50	6.87E−06	NA	NA	T
29	SUFU	10:102503962–102633457	0.08	5.25	1.50E−07	0.15	0.85	0.42	6.78E−01	NA	NA	F
30	WBP1L	10:102743970–102816264	0.40	4.77	1.82E−06	1.00	0.00	0.97	3.33E−01	NA	NA	T
31	SYT13	11:45240302–45286333	0.45	−4.86	1.15E−06	0.03	0.97	−2.79	5.34E−03	NA	NA	T
32	MYO7A	11:77128264–77215241	0.12	5.11	3.24E−07	0.99	0.00	3.01	2.61E−03	NA	NA	T
33	AAMDC	11:77821162–77872352	0.05	−4.74	2.18E−06	0.26	0.56	−1.00	3.16E−01	NA	NA	T
34	NABP2	12:56224341–56229854	0.07	−6.01	1.90E−09	0.63	0.03	−4.49	7.01E−06	NA	NA	T
35	SPPL3	12:120762510–120904352	0.19	7.51	5.98E−14	0.97	0.03	2.81	4.99E−03	NA	NA	F
36	ABCB9	12:122920951–122966509	0.14	−4.74	2.18E−06	0.95	0.05	−0.76	4.45E−01	NA	NA	F
37	PITPNM2	12:122983480–123110489	0.15	−5.29	1.19E−07	0.81	0.19	−1.00	3.20E−01	NA	NA	T
38	CDK2AP1	12:123260970–123272316	0.44	5.31	1.09E−07	0.47	0.53	0.90	3.70E−01	NA	NA	F
39	CHP1	15:41231239–41281885	0.33	−6.05	1.42E−09	0.79	0.21	−2.76	5.73E−03	NA	NA	F
40	ITPKA	15:41493858–41503559	0.21	5.10	3.39E−07	0.01	0.99	2.71	6.72E−03	NA	NA	T
41	IQGAP1	15:90388241–90502243	0.41	−5.29	1.23E−07	0.01	0.99	−1.09	2.76E−01	NA	NA	F
42	DEXI	16:10928891–10942400	0.32	−7.43	1.08E−13	1.00	0.00	−2.79	5.28E−03	NA	NA	T
43	NSMCE1	16:27224994–27268792	0.26	7.14	9.51E−13	1.00	0.00	3.04	2.40E−03	NA	NA	T
44	IL4R	16:27313909–27364778	0.17	4.83	1.37E−06	0.66	0.33	2.08	3.78E−02	NA	NA	F
45	ATP2A1	16:28878488–28904509	0.08	−4.65	3.30E−06	0.29	0.67	−0.97	3.34E−01	NA	NA	T
46	LINC00672	17:38925168–38929384	0.10	6.88	6.07E−12	0.62	0.01	−1.33	1.83E−01	NA	NA	T
47	RPL19	17:39200283–39204727	0.15	−8.36	6.03E−17	0.37	0.20	1.48	1.39E−01	NA	NA	T
48	MED1	17:39404285–39451274	0.17	8.22	2.11E−16	1.00	0.00	−0.16	8.73E−01	NA	NA	T
49	STARD3	17:39637080–39664201	0.35	14.75	3.30E–49	1.00	0.00	2.05	4.07E−02	NA	NA	F
50	PGAP3	17:39671122–39688070	0.61	15.20	3.51E–52	1.00	0.00	1.50	1.34E−01	NA	NA	F
51	ERBB2	17:39688084–39728662	0.32	13.56	7.18E–42	1.00	0.00	0.61	5.42E−01	NA	NA	F
52	GRB7	17:39737909–39747285	0.09	9.97	2.15E–23	0.99	0.00	−2.54	1.11E−02	NA	NA	T
53	IKZF3	17:39757715–39864188	0.11	−21.88	3.95E−106	0.42	0.58	−2.90	3.69E−03	NA	NA	T
54	GSDMB	17:39904595–39918650	0.52	23.66	9.07E−124	0.01	0.99	2.70	6.88E−03	NA	NA	F
55	GSDMA	17:39962973–39977766	0.34	−13.36	1.02E–40	1.00	0.00	−2.91	3.62E−03	NA	NA	F
56	PSMD3	17:39980768–39997960	0.06	11.34	8.19E–30	1.00	0.00	2.35	1.87E−02	NA	NA	T
57	MED24	17:40019097–40054636	0.45	5.49	4.12E−08	1.00	0.00	0.02	9.81E−01	NA	NA	F
58	RARA-AS1	17:40340867–40343136	0.06	6.28	3.33E−10	0.98	0.01	1.73	8.38E−02	NA	NA	T
59	SMARCE1	17:40627728–40647851	0.50	−4.71	2.46E−06	1.00	0.00	−2.02	4.32E−02	NA	NA	F
60	STAT5A	17:42287547–42311942	0.13	−4.93	8.26E−07	0.71	0.28	−3.43	6.09E−04	NA	NA	T
61	HEXIM1	17:45147317–45152101	0.27	−5.74	9.54E−09	1.00	0.00	−1.26	2.09E−01	NA	NA	T
62	SPATA32	17:45254393–45262112	0.73	−5.16	2.46E−07	0.98	0.02	−2.72	6.45E−03	NA	NA	F
63	LRRC37A4P	17:45505883–45520523	0.32	5.67	1.43E−08	1.00	0.00	0.17	8.62E−01	NA	NA	F
64	LOC644172	17:45600103–45601862	0.10	−5.58	2.34E−08	1.00	0.00	−0.47	6.40E−01	NA	NA	T
65	MGC57346	17:45620329–45637963	0.22	−5.51	3.58E−08	1.00	0.00	−0.38	7.02E−01	NA	NA	T
66	CRHR1-IT1	17:45638975–45646229	0.26	−5.62	1.90E−08	1.00	0.00	−0.39	6.93E−01	NA	NA	F
67	KANSL1	17:46029916–46225374	0.14	−5.07	3.90E−07	1.00	0.00	−0.24	8.08E−01	NA	NA	F
68	KANSL1-AS1	17:46193573–46196723	0.29	−5.13	2.87E−07	1.00	0.00	−0.10	9.23E−01	NA	NA	F
69	LRRC37A	17:46295131–46337794	0.40	−6.04	1.58E−09	1.00	0.01	−0.15	8.82E−01	NA	NA	F
70	ZNF652	17:49289206–49362473	0.46	−5.73	1.02E−08	0.97	0.03	−3.30	9.61E−04	NA	NA	T
71	SPOP	17:49598884–49678163	0.08	5.04	4.72E−07	0.60	0.02	4.06	4.81E−05	NA	NA	T
72	SMAD4	18:51030213–51085041	0.25	−6.29	3.26E−10	0.06	0.94	−2.60	9.22E−03	NA	NA	F
73	POLI	18:54269479–54298234	0.52	6.28	3.49E−10	0.41	0.60	3.07	2.17E−03	NA	NA	F
74	MUC16	19:8848844–8981342	0.25	−6.55	5.91E−11	0.04	0.96	−2.56	1.05E−02	NA	NA	T
75	FOXA3	19:45864260–45873797	0.42	5.12	3.10E−07	0.02	0.97	4.26	2.09E−05	NA	NA	T
76	STMN3	20:63639705–63653610	0.23	−5.23	1.65E−07	0.03	0.97	−3.36	7.75E−04	NA	NA	F
77	LIME1	20:63735701–63739107	0.18	4.68	2.82E−06	0.71	0.28	3.07	2.14E−03	NA	NA	T
**Shared**												
78	IL18R1	2:102356283–102398775	0.08	−9.22	2.92E–20	0.54	0.05	−5.37	7.70E−08	0.56	0.02	F
79	D2HGDH	2:241734579–241768816	0.64	10.37	3.24E–25	1.00	0.00	6.74	1.56E−11	1.00	0.00	F
80	GAL3ST2	2:241776825–241804287	0.43	9.07	1.20E−19	1.00	0.00	6.82	9.00E−12	1.00	0.00	T
81	TSLP	5:111070080–111078024	0.34	14.69	7.43E–49	1.00	0.00	10.20	1.96E–24	1.00	0.00	F
82	PDLIM4	5:132257658–132273454	0.17	7.20	5.84E−13	1.00	0.00	7.87	3.47E−15	0.99	0.01	F
83	BACH2	6:89926528–90296908	0.09	5.79	7.00E−09	0.14	0.84	5.17	2.29E−07	0.14	0.84	T
84	IL33	9:6215786–6257983	0.25	18.85	2.80E–79	0.00	1.00	12.37	3.68E–35	0.01	0.99	T
85	RAB5B	12:55973913–55996683	0.05	6.31	2.85E−10	0.42	0.15	6.22	4.84E−10	0.07	0.86	T
86	RPS26	12:56041902–56044223	0.33	6.95	3.78E−12	1.00	0.00	4.61	3.97E−06	1.00	0.00	F
87	UBAC2-AS1	13:99196374–99200757	0.09	5.96	2.50E−09	0.14	0.86	5.50	3.79E−08	0.28	0.72	T
88	UBAC2	13:99200425–99386499	0.29	−6.78	1.18E−11	0.04	0.96	−5.14	2.68E−07	0.20	0.80	T
**Adult Onset Asthma**												
89	RERE	Chr1:8352404–8817640	0.14	4.18	2.92E−05	NA	NA	4.74	2.13E−06	0.02	0.98	F
90	C7orf26	Chr7:6590021–6608726	0.06	2.05	4.04E−02	NA	NA	4.94	7.88E−07	0.23	0.47	T
91	MUC2	Chr11:1074875–1110508	0.21	−2.13	3.34E−02	NA	NA	−5.16	2.47E−07	0.02	0.98	T
92	MUC5AC	Chr11:1157953–1201141	0.32	2.70	6.90E−03	NA	NA	7.23	4.70E−13	0.02	0.98	T
93	FADS1	Chr11:61799625–61817057	0.32	1.58	1.13E−01	NA	NA	6.48	9.03E−11	0.04	0.96	T
94	SIK2	Chr11:111602449–111730855	0.27	−2.65	8.05E−03	NA	NA	−5.12	3.11E−07	0.21	0.79	F
95	HDAC7	Chr12:47782711–47819980	0.16	−0.98	3.27E−01	NA	NA	−4.65	3.30E−06	0.39	0.60	T

The original version of the article contained errors in Supplementary Data 1,2,3,4 and 9. The HTML has been updated to include a corrected version of these Supplementary Data files; the original incorrect versions of these Supplementary Data files can be found associated with this Correction.

## Supplementary information

Incorrect Supplementary Data 1-14

