# Supplementary material for: Nasal airway transcriptome-wide association study of asthma reveals genetically driven mucus pathobiology
Source: Nat Commun. 2022 Mar 28;13:1632. doi: 10.1038/s41467-022-28973-7 (PMC8960819; doi:10.1038/s41467-022-28973-7)
Supplement: Supplementary file 1 — Supplementary Information [file 41467_2022_28973_MOESM1_ESM.pdf]

Supplementary Information for:

Nasal airway epithelial TWAS of childhood asthma reveals novel, genetically driven mucus pathobiology

Authors: Satria P. Sajuthi[1], Jamie L. Everman[1], Nathan D. Jackson[1], Benjamin Saef[1], Cydney L. Rios[1], Camille M. Moore[1,2,3], Angel CY Mak[4], Celeste Eng[4], Ana Fairbanks-Mahnke[1], Sandra Salazar[4], Jennifer Elhawary[4], Scott Hunstman[4], Vivian Medina[5], Deborah A. Nickerson[6], Soren Germer[7], Michael C. Zody[7], Gonçalo Abecasis[8], Hyun Min Kang[8], Kenneth M. Rice[9], Rajesh Kumar[10], Noah A. Zaitlen[11], Sam Oh[4], NHLBI Trans-Omics for Precision Medicine (TOPMed) Consortium*, José Rodríguez-Santana[5], Esteban G. Burchard[4,12], and Max A. Seibold[1,13,14]

Affiliations:

[1]Center for Genes, Environment, and Health, National Jewish Health, Denver, CO;

[2]Department of Biomedical Research, National Jewish Health, Denver, CO;

[3]Department of Biostatistics and Informatics, University of Colorado, Denver, CO;

[4]Department of Medicine, University of California-San Francisco, San Francisco, CA;

[5]Centro de Neumología Pediátrica, San Juan, Puerto Rico;

[6]Department of Genome Sciences, University of Washington, Seattle, WA;

[7]New York Genome Center, New York, NY;

[8]Center for Statistical Genetics, University of Michigan, Ann Arbor, MI;

[9]Department of Biostatistics, University of Washington, Seattle, WA;

[10]Ann and Robert H. Lurie Children's Hospital of Chicago, Department of Pediatrics, Northwestern University, Chicago, IL;

[11]Department of Neurology and Computational Medicine, University of California Los Angeles, Los Angeles, CA;

[12]Department of Bioengineering and Therapeutic Sciences, University of California-San Francisco, San Francisco, CA;

[13]Department of Pediatrics, National Jewish Health, Denver, CO;

[14]Division of Pulmonary Sciences and Critical Care Medicine, University of Colorado School of Medicine, Aurora, CO;

*A list of authors and their affiliations appears at the end of the paper.

Corresponding Author:

Max A. Seibold, Ph.D.

Professor of Pediatrics

Center for Genes, Environment, and Health

National Jewish Health

1400 Jackson St.

Denver, CO 80206

Phone (303) 270-2544

Fax (303) 270-2136

seiboldm@njhealth.org

Supplementary Figure 1

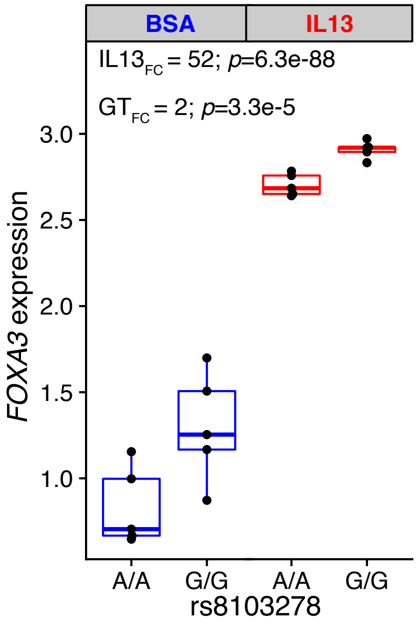

Supplementary Figure 1. *FOXA3* expression is regulated by IL13 and rs8103278 variant.

Box plots of log normalized *FOXA3* expression stratified by IL13 treatment and genotype of rs8103278 (A/A, *n*=5; G/G, *n*=5). Box centers give the median, upper and lower box bounds correspond to first and third quartiles, and the upper/lower whiskers extend from the upper/lower bounds up to/down from the largest/smallest value, no further than 1.5 × IQR from the upper/lower bound (where IQR is the inter-quartile range). Data beyond the end of whiskers are plotted individually. Two sided p-values were obtained from DESeq2.

TOPMed Members

Namiko Abe[1], Francois Aguet[2], Christine Albert[3], Laura Almasy[4], Alvaro Alonso[5], Seth Ament[6], Peter Anderson[7], Pramod Anugu[8], Deborah Applebaum-Bowden[9], Kristin Ardlie[2], Dan Arking[10], Donna K Arnett[11], Allison Ashley-Koch[12], Stella Aslibekyan[13], Tim Assimes[14], Paul Auer[15], Dimitrios Avramopoulos[10], Najib Ayas[16], Adithya Balasubramanian[17], John Barnard[18], Kathleen Barnes[19], R. Graham Barr[20], Emily Barron-Casella[10], Lucas Barwick[21], Terri Beaty[10], Gerald Beck[18], Diane Becker[10], Lewis Becker[10], Rebecca Beer[22], Amber Beitelshees[6], Emelia Benjamin[23], Takis Benos[24], Marcos Bezerra[25], Larry Bielak[26], Joshua Bis[7], Thomas Blackwell[26], John Blangero[27], Eric Boerwinkle[28], Donald W. Bowden[29], Russell Bowler[30], Jennifer Brody[7], Ulrich Broeckel[15], Jai Broome[7], Deborah Brown[28], Karen Bunting[1], Carlos Bustamante[14], Erin Buth[7], Brian Cade[31], Jonathan Cardwell[32], Vincent Carey[31], Julie Carrier[33], April Carson[8], Cara Carty[34], Richard Casaburi[35], Juan P Casas Romero[31], James Casella[10], Peter Castaldi[31], Mark Chaffin[2], Christy Chang[6], Yi-Cheng Chang[36], Daniel Chasman[31], Sameer Chavan[32], Bo-Juen Chen[1], Wei-Min Chen[37], Yii-Der Ida Chen[38], Michael Cho[31], Seung Hoan Choi[2], Lee-Ming Chuang[36], Mina Chung[18], Ren-Hua Chung[39], Clary Clish[2], Suzy Comhair[18], Matthew Conomos[7], Elaine Cornell[40], Adolfo Correa[8], Carolyn Crandall[35], James Crapo[30], L. Adrienne Cupples[41], Joanne Curran[27], Jeffrey Curtis[26], Brian Custer[42], Coleen Damcott[6], Dawood Darbar[43], Sean David[44], Colleen Davis[7], Michelle Daya[32], Mariza de Andrade[45], Lisa de las Fuentes[46], Paul de Vries[28], Michael DeBaun[47], Ranjan Deka[48], Dawn DeMeo[31], Scott Devine[6], Huyen Dinh[17], Harsha Doddapaneni[17], Qing Duan[49], Shannon Dugan-Perez[17], Ravi Duggirala[27], Jon Peter Durda[40], Susan K. Dutcher[46], Charles Eaton[50], Lynette Ekunwe[8], Adel El Boueiz[51], Patrick Ellinor[52], Leslie Emery[7], Serpil Erzurum[18], Charles Farber[37], Jesse Farek[17], Tasha Fingerlin[30], Matthew Flickinger[26], Myriam Fornage[28], Nora Franceschini[49], Chris Frazar[7], Mao Fu[6], Stephanie M. Fullerton[7], Lucinda Fulton[46], Stacey Gabriel[2], Weiniu Gan[22], Shanshan Gao[32], Yan Gao[8], Margery Gass[53], Heather Geiger[1], Bruce Gelb[54], Mark Geraci[24], Robert Gerszten[55], Auyon Ghosh[31], Richard Gibbs[17], Chris Gignoux[14], Mark Gladwin[24], David Glahn[56], Stephanie Gogarten[7], Da-Wei Gong[6], Harald Goring[27], Sharon Graw[57], Kathryn J. Gray[58], Daniel Grine[32], Colin Gross[26], C. Charles Gu[46], Yue Guan[6], Xiuqing Guo[38], Namrata Gupta[2], David M. Haas[59], Jeff Haessler[53], Michael Hall[8], Yi Han[17], Patrick Hanly[60], Daniel Harris[6], Nicola L. Hawley[61], Jiang He[62], Ben Heavner[7], Susan Heckbert[7], Ryan Hernandez[63], David Herrington[29], Craig Hersh[31], Bertha Hidalgo[13], James Hixson[28], Brian Hobbs[31], John Hokanson[32], Elliott Hong[6], Karin Hoth[64], Chao (Agnes) Hsiung[39], Jianhong Hu[17], Yi-Jen Hung[65], Haley Huston[66], Chii Min Hwu[67], Marguerite Ryan Irvin[13], Rebecca Jackson[68], Deepti Jain[7], Cashell Jaquish[22], Jill Johnsen[66], Andrew Johnson[22], Craig Johnson[7], Rich Johnston[5], Kimberly Jones[10], Robert Kaplan[69], Sharon Kardia[26], Shannon Kelly[63], Eimear Kenny[54], Michael Kessler[6], Alyna Khan[7], Ziad Khan[17], Wonji Kim[51], John Kimoff[70], Greg Kinney[32], Barbara Konkle[66], Charles Kooperberg[53], Holly Kramer[71], Christoph Lange[72], Ethan Lange[32], Leslie Lange[32], Cathy Laurie[7], Cecelia Laurie[7], Meryl LeBoff[31], Jiwon Lee[31], Sandra Lee[17], Wen-Jane Lee[67], Jonathon LeFaive[26], David Levine[7], Dan Levy[22], Joshua Lewis[6], Xiaohui Li[38], Yun Li[49], Henry Lin[38], Honghuang Lin[41], Xihong Lin[72], Simin Liu[50], Yongmei Liu[12], Yu Liu[14], Ruth J.F. Loos[54], Steven Lubitz[52], Kathryn Lunetta[41], James Luo[22], Ulysses Magalang[73], Michael Mahaney[27], Barry Make[10], Ani Manichaikul[37], Alisa Manning[74], JoAnn Manson[31], Lisa Martin[75], Melissa Marton[1], Susan Mathai[32], Rasika Mathias[10], Susanne May[7], Patrick McArdle[6], Merry-Lynn McDonald[13], Sean McFarland[51], Stephen McGarvey[50], Daniel McGoldrick[7], Caitlin McHugh[7], Becky McNeil[76], Hao Mei[8], James Meigs[52], Vipin Menon[17], Luisa Mestroni[57], Ginger Metcalf[17], Deborah A Meyers[77], Emmanuel Mignot[14], Julie Mikulla[22], Nancy Min[8], Mollie Minear[78], Ryan L Minster[24], Braxton D. Mitchell[6], Matt Moll[31], Zeineen Momin[17], May E. Montasser[6], Courtney Montgomery[79], Donna Muzny[17], Josyf C Mychaleckyj[37], Girish Nadkarni[54], Rakhi Naik[10], Take Naseri[80], Pradeep Natarajan[2], Sergei Nekhai[81], Sarah C. Nelson[7], Bonnie Neltner[32], Caitlin Nessner[17], Osuji Nkechinyere[17], Kari North[49], Jeff O'Connell[6], Tim O'Connor[6], Heather Ochs-Balcom[82], Geoffrey Okwuonu[17], Allan Pack[83], David T. Paik[14], Nicholette Palmer[29], James Pankow[84], George

Papanicolaou[22], Cora Parker[76], Gina Peloso[41], Juan Manuel Peralta[27], Marco Perez[14], James Perry[6], Ulrike Peters[53], Patricia Peyser[26], Lawrence S Phillips[5], Jacob Pleiness[26], Toni Pollin[6], Wendy Post[10], Julia Powers Becker[32], Meher Preethi Boorgula[32], Michael Preuss[54], Bruce Psaty[7], Pankaj Qasba[22], Dandi Qiao[31], Zhaohui Qin[5], Nicholas Rafaels[32], Laura Raffield[49], Mahitha Rajendran[17], Vasan S. Ramachandran[41], D.C. Rao[46], Laura Rasmussen-Torvik[85], Aakrosh Ratan[37], Susan Redline[31], Robert Reed[6], Catherine Reeves[1], Elizabeth Regan[30], Alex Reiner[86], Muagututi'a Sefuiva Reupena[87], Stephen Rich[37], Rebecca Robillard[88], Nicolas Robine[1], Dan Roden[47], Carolina Roselli[2], Jerome Rotter[38], Ingo Ruczinski[10], Alexi Runnels[1], Pamela Russell[32], Sarah Ruuska[66], Kathleen Ryan[6], Ester Cerdeira Sabino[89], Danish Saleheen[20], Shabnam Salimi[6], Sejal Salvi[17], Steven Salzberg[10], Kevin Sandow[38], Vijay G. Sankaran[51], Jireh Santibanez[17], Karen Schwander[46], David Schwartz[32], Frank Sciurba[24], Christine Seidman[90], Jonathan Seidman[90], Frédéric Sériès[91], Vivien Sheehan[5], Stephanie L. Sherman[5], Amol Shetty[6], Aniket Shetty[32], Wayne Hui-Heng Sheu[67], M. Benjamin Shoemaker[47], Brian Silver[92], Edwin Silverman[31], Robert Skomro[93], Albert Vernon Smith[26], Jennifer Smith[26], Josh Smith[7], Nicholas Smith[7], Tanja Smith[1], Sylvia Smoller[69], Beverly Snively[29], Michael Snyder[14], Tamar Sofer[31], Nona Sotoodehnia[7], Adrienne M. Stilp[7], Garrett Storm[32], Elizabeth Streeten[6], Jessica Lasky Su[31], Yun Ju Sung[46], Jody Sylvia[31], Adam Szpiro[7], Daniel Taliun[26], Hua Tang[14], Margaret Taub[10], Kent D. Taylor[38], Matthew Taylor[57], Simeon Taylor[6], Marilyn Telen[12], Timothy A. Thornton[7], Machiko Threlkeld[7], Lesley Tinker[53], David Tirschwell[7], Sarah Tishkoff[83], Hemant Tiwari[13], Catherine Tong[7], Russell Tracy[40], Michael Tsai[84], Dhananjay Vaidya[10], David Van Den Berg[94], Peter VandeHaar[26], Scott Vrieze[84], Tarik Walker[32], Robert Wallace[64], Avram Walts[32], Fei Fei Wang[7], Heming Wang[95], Jiongming Wang[26], Karol Watson[35], Jennifer Watt[17], Daniel E. Weeks[24], Joshua Weinstock[26], Bruce Weir[7], Scott T Weiss[31], Lu-Chen Weng[52], Jennifer Wessel[59], Cristen Willer[26], Kayleen Williams[7], L. Keoki Williams[96], Carla Wilson[31], James Wilson[55], Lara Winterkorn[1], Quenna Wong[7], Joseph Wu[14], Huichun Xu[6], Lisa Yanek[10], Ivana Yang[32], Ketian Yu[26], Seyedeh Maryam Zekavat[2], Yingze Zhang[24], Snow Xueyan Zhao[30], Wei Zhao[26], Xiaofeng Zhu[97], Sebastian Zoellner[26]

1 - New York Genome Center; 2 - Broad Institute; 3 - Cedars Sinai; 4 - Children's Hospital of Philadelphia, University of Pennsylvania; 5 - Emory University; 6 - University of Maryland; 7 - University of Washington; 8 - University of Mississippi; 9 - National Institutes of Health; 10 - Johns Hopkins University; 11 - University of Kentucky; 12 - Duke University; 13 - University of Alabama; 14 - Stanford University; 15 - Medical College of Wisconsin; 16 - Providence Health Care; 17 - Baylor College of Medicine Human Genome Sequencing Center; 18 - Cleveland Clinic; 19 - Tempus, University of Colorado Anschutz Medical Campus; 20 - Columbia University; 21 - The Emmes Corporation; 22 - National Heart, Lung, and Blood Institute, National Institutes of Health; 23 - Boston University, Massachusetts General Hospital; 24 - University of Pittsburgh; 25 - Fundação de Hematologia e Hemoterapia de Pernambuco - Hemope; 26 - University of Michigan; 27 - University of Texas Rio Grande Valley School of Medicine; 28 - University of Texas Health at Houston; 29 - Wake Forest Baptist Health; 30 - National Jewish Health; 31 - Brigham & Women's Hospital; 32 - University of Colorado at Denver; 33 - University of Montreal; 34 - Washington State University; 35 - University of California, Los Angeles; 36 - National Taiwan University; 37 - University of Virginia; 38 - Lundquist Institute; 39 - National Health Research Institute Taiwan; 40 - University of Vermont; 41 - Boston University; 42 - Vitalant Research Institute; 43 - University of Illinois at Chicago; 44 - University of Chicago; 45 - Mayo Clinic; 46 - Washington University in St Louis; 47 - Vanderbilt University; 48 - University of Cincinnati; 49 - University of North Carolina; 50 - Brown University; 51 - Harvard University; 52 - Massachusetts General Hospital; 53 - Fred Hutchinson Cancer Research Center; 54 - Icahn School of Medicine at Mount Sinai; 55 - Beth Israel Deaconess Medical Center; 56 - Boston Children's Hospital, Harvard Medical School; 57 - University of Colorado Anschutz Medical Campus; 58 - Mass General Brigham; 59 - Indiana University; 60 - University of Calgary; 61 - Yale University; 62 - Tulane University; 63 - University of California, San Francisco; 64 - University of Iowa; 65 - Tri-Service General Hospital National Defense Medical Center; 66 - Blood Works Northwest; 67 - Taichung Veterans General Hospital Taiwan; 68 - Oklahoma State University Medical Center; 69 - Albert Einstein College of Medicine; 70 - McGill University; 71 - Loyola University; 72 - Harvard School of Public Health; 73 - Ohio State University; 74 - Broad Institute, Harvard University, Massachusetts General Hospital; 75 - George Washington University; 76 -

RTI International; 77 - University of Arizona; 78 - National Institute of Child Health and Human Development, National Institutes of Health; 79 - Oklahoma Medical Research Foundation; 80 - Ministry of Health, Government of Samoa; 81 - Howard University; 82 - University at Buffalo; 83 - University of Pennsylvania; 84 - University of Minnesota; 85 - Northwestern University; 86 - Fred Hutchinson Cancer Research Center, University of Washington; 87 - Lutia I Puava Ae Mapu I Fagalele; 88 - University of Ottawa; 89 - Universidade de Sao Paulo; 90 - Harvard Medical School; 91 - Université Laval; 92 - UMass Memorial Medical Center; 93 - University of Saskatchewan; 94 - University of Southern California; 95 - Brigham & Women's Hospital, Mass General Brigham; 96 - Henry Ford Health System; 97 - Case Western Reserve University
