# Supplementary material for: Nasal airway transcriptome-wide association study of asthma reveals genetically driven mucus pathobiology
Source: Nat Commun. 2022 Mar 28;13:1632. doi: 10.1038/s41467-022-28973-7 (PMC8960819; doi:10.1038/s41467-022-28973-7)
Supplement: Supplementary file 3 — Description of Additional Supplementary Files [file 41467_2022_28973_MOESM3_ESM.pdf]

Description of Additional Supplementary Files

File Name: Supplementary Data 1
Description: Table of independent eVariants. Two-sided p-values were obtained from QTLtools.

File Name: Supplementary Data 2
Description: Table of eGenes. Two-sided p-values were obtained from FASTQTL. Benjamini-Hochberg correction was used to control for false discovery rate.

File Name: Supplementary Data 3
Description: Table of COA and AOA GWAS loci from Ferreira et. al. (2019) annotated with TWAS genes in proximity

File Name: Supplementary Data 4
Description: Table of COA and AOA TWAS genes from nasal epithelium and six other GTEX tissues

File Name: Supplementary Data 5
Description: Table of linear regression coefficient and its corresponding p-value for predicting MUC5AC expression. Two sided P-values were obtained by fitting linear regression model with lm()

File Name: Supplementary Data 6
Description: Trans eQTL table of rs12788104. Two-sided p-values were obtained from limma. Benjamini-Hochberg correction was used to control for false discovery rate.

File Name: Supplementary Data 7
Description: Table of enriched terms from rs12788104 trans-eQTL genes. One sided enrichment p-values were obtained from Enrichr. Benjamini-Hochberg correction was used to control for false discovery rate.

File Name: Supplementary Data 8
Description: Table of enriched gene networks from rs12788104 and rs8103278 trans-eQTL genes. One sided p-values were obtained using hypergeometric test to test for gene set enrichment.

File Name: Supplementary Data 9
Description: Cough (UKBB_22504) and Phlegm (UKBB_22502) nasal TWAS hits. Two sided TWAS p-values were obtained from FUSION.

File Name: Supplementary Data 10
Description: Table of linear regression coefficient and its corresponding p-value for predicting FOXA3 expression. Two-sided p-values were obtained by fitting linear regression model with lm()

File Name: Supplementary Data 11
Description: Table of logistic regression coefficient and its corresponding p-value for predicting asthma risk. Two-sided p-values were obtained by fitting logistic regression model using glm()

File Name: Supplementary Data 12
Description: Trans eQTL table of rs8103278. Two-sided p-values were obtained from limma. Benjamini-Hochberg correction was used to control for false discovery rate.

File Name: Supplementary Data 13
Description: Clinical and basic demographic information of GALA II participants.

File Name: Supplementary Data 14
Description: T2 network genes.
