# Supplementary material for: Nasal airway transcriptome-wide association study of asthma reveals genetically driven mucus pathobiology
Source: Nat Commun. 2022 Mar 28;13:1632. doi: 10.1038/s41467-022-28973-7 (PMC8960819; doi:10.1038/s41467-022-28973-7)
Supplement: Supplementary file 5 — Reporting Summary [file 41467_2022_28973_MOESM5_ESM.pdf]

nature portfolio

Corresponding author(s): Max A. Seibold

Last updated by author(s): Jan 25, 2022

Reporting Summary

Nature Portfolio wishes to improve the reproducibility of the work that we publish. This form provides structure for consistency and transparency in reporting. For further information on Nature Portfolio policies, see our Editorial Policies and the Editorial Policy Checklist.

Statistics

For all statistical analyses, confirm that the following items are present in the figure legend, table legend, main text, or Methods section.

n/a	Confirmed	
☐	☒	The exact sample size (*n*) for each experimental group/condition, given as a discrete number and unit of measurement
☐	☒	A statement on whether measurements were taken from distinct samples or whether the same sample was measured repeatedly
☐	☒	The statistical test(s) used AND whether they are one- or two-sided<br>*Only common tests should be described solely by name; describe more complex techniques in the Methods section.*
☐	☒	A description of all covariates tested
☐	☒	A description of any assumptions or corrections, such as tests of normality and adjustment for multiple comparisons
☐	☒	A full description of the statistical parameters including central tendency (e.g. means) or other basic estimates (e.g. regression coefficient) AND variation (e.g. standard deviation) or associated estimates of uncertainty (e.g. confidence intervals)
☐	☒	For null hypothesis testing, the test statistic (e.g. *F*, *t*, *r*) with confidence intervals, effect sizes, degrees of freedom and *P* value noted<br>*Give P values as exact values whenever suitable.*
☒	☐	For Bayesian analysis, information on the choice of priors and Markov chain Monte Carlo settings
☒	☐	For hierarchical and complex designs, identification of the appropriate level for tests and full reporting of outcomes
☐	☒	Estimates of effect sizes (e.g. Cohen's *d*, Pearson's *r*), indicating how they were calculated

Our web collection on statistics for biologists contains articles on many of the points above.

Software and code

Policy information about availability of computer code

Data collection	None
Data analysis	Skewer version 0.2.2 was used for trimming bulk RNA-seq data;<br>GSNAP version 2016_05_01 was used for mapping bulk RNA-seq data from GALA II;<br>HISAT2 version 2.1.0 was used for mapping bulk RNA-seq data from ALI cultures;<br>HTSeq-count version 0.9.1 was used for bulk-RNA seq data gene quantification;<br>DESeq2 version 1.22.2 was used to normalize bulk RNA-seq expression data and differential expression;<br>WGCNA version 1.68 was used to perform network analysis;<br>edgeR version 3.22.3 was used to perform TMM normalization;<br>ADMIXTURE version 1.3 was used on genotype data to compute admixture factors;<br>PEER version 1.3 was used to generate PEER factors;<br>GCTA version 1.26 was used to estimate gene expression heritabilities;<br>FastQTL version 2.184 was used to perform cis-eQTL analysis;<br>QTLTools version 1.1 was used to perform stepwise regression;<br>FUSION/TWAS version 1.0 was used to perform TWAS analysis;<br>Limma version 3.46 was used to perform trans-eQTL analysis of rs8103278 and rs12788104;<br>enrichR version 3.0 was used for functional enrichment analysis;<br>glmmTMB version 1.0.2.1 was used to perform analysis on MUC5AC immunofluorescence study;<br>lme4 version 1.1.26 and lmerTest version 3.1.3 were used to perform analysis on MUC5AC protein assay study;<br>JASPAR2020 version 0.99.10 was used to download Motif logo;<br>TFBSTools version 1.28.0 was used to compute Motif information content matrix;<br>R version 3.4.1 was used for analysis and visualization;

Custom scripts used to perform the analyses described in the paper have been deposited to the github repository [https://github.com/seiboldlab/Nasal_TWAS]

For manuscripts utilizing custom algorithms or software that are central to the research but not yet described in published literature, software must be made available to editors and reviewers. We strongly encourage code deposition in a community repository (e.g. GitHub). See the Nature Portfolio guidelines for submitting code & software for further information.

Data

Policy information about availability of data

All manuscripts must include a data availability statement. This statement should provide the following information, where applicable:

- Accession codes, unique identifiers, or web links for publicly available datasets
- A description of any restrictions on data availability
- For clinical datasets or third party data, please ensure that the statement adheres to our policy

GALA II RNA-seq data used in this study have been previously deposited in the National Center for Biotechnology Information/Gene Expression Omnibus (GEO) GSE152004 [https://www.ncbi.nlm.nih.gov/geo/query/acc.cgi?acc=GSE152004];
The processed protein and RNAseq data used in the invitro experiments are available at the github repository [https://github.com/seiboldlab/Nasal_TWAS/tree/main/Data];
GO Biological Process 2018 table [https://maayanlab.cloud/Enrichr/geneSetLibrary?mode=text&libraryName=GO_Biological_Process_2018];
GO Molecular Function 2018 table [https://maayanlab.cloud/Enrichr/geneSetLibrary?mode=text&libraryName=GO_Molecular_Function_2018];
GO Cellular Component 2018 table [https://maayanlab.cloud/Enrichr/geneSetLibrary?mode=text&libraryName=GO_Cellular_Component_2018];
Ligand Perturbations from GEO up table [https://maayanlab.cloud/Enrichr/geneSetLibrary?mode=text&libraryName=Ligand_Perturbations_from_GEO_up];
Kyoto Encyclopedia of Genes and Genomes 2019 Human table [https://maayanlab.cloud/Enrichr/geneSetLibrary?mode=text&libraryName=KEGG_2019_Human];
Reactome 2016 table [https://maayanlab.cloud/Enrichr/geneSetLibrary?mode=text&libraryName=Reactome_2016];
Cell type marker gene sets were obtained from Supplemental Table S4 in Travaglini, et al. [https://static-content.springer.com/esm/art%3A10.1038%2Fs41586-020-2922-4/MediaObjects/41586_2020_2922_MOESM6_ESM.xlsx];
TOPMed freeze 8 variant calls are available from dbGaP accession phs000920.v2.p2 [https://www.ncbi.nlm.nih.gov/projects/gap/cgi-bin/study.cgi?study_id=phs000920.v4.p2];
CADD v1.5 database [https://cadd.gs.washington.edu];
JASPAR 2020 database [http://jaspar.genereg.net];
Ferreira et. al. UKBB COA GWAS summary statistics [https://genepi.qimr.edu.au/staff/manuelF/gwas_results/CHILD_ONSET_ASTHMA.20180501.allchr.assoc.GC.gz];
Ferreira et. al. UKBB AOA GWAS summary statistics [https://genepi.qimr.edu.au/staff/manuelF/gwas_results/ADULT1_ADULT2_ONSET_ASTHMA.20180716.allchr.assoc.GC.gz];
UKBB cough (22502) GWAS summary statistics [https://broad-ukb-sumstats-us-east-1.s3.amazonaws.com/round2/additive-tsvs/22502.gwas.imputed_v3.both_sexes.tsv.bgz]
UKBB phlegm (22504) GWAS summary statistics [https://broad-ukb-sumstats-us-east-1.s3.amazonaws.com/round2/additive-tsvs/22504.gwas.imputed_v3.both_sexes.tsv.bgz]

Field-specific reporting

Please select the one below that is the best fit for your research. If you are not sure, read the appropriate sections before making your selection.

☒ Life sciences ☐ Behavioural & social sciences ☐ Ecological, evolutionary & environmental sciences

For a reference copy of the document with all sections, see nature.com/documents/nr-reporting-summary-flat.pdf

Life sciences study design

All studies must disclose on these points even when the disclosure is negative.

Sample size

For GALA II nasal transcriptomic study, no formal sample size calculation was done. The analysis from from this dataset was used to describe the heterogeneity of nasal airway transcriptomes. There are 695 donors with RNA-seq data.

For GALA II eQTL study, no formal sample size calculation was done. There are 681 donors with paired genotype and expression data which is larger than the sample size of any single tissue in GTEx.

For the analysis of FOXA3 RNA-seq expression data of GALA donors between paired IL13-stimulated and control samples (rs8103278-GG, N=5 donors; rs8103278-AA, N=5 donors), no formal sample size calculation was done. This sample size was sufficient to identify FOXA3 expression changes by genotype and treatment.

For the analysis of MUC5AC ELISA assay data (rs12788104-GG, N=5 donors; rs12788104-AA, N=5 donors), no formal sample size calculation was done. This sample size was sufficient to identify changes in MUC5AC protein level.

For the MUC5AC immunofluorescence study (rs12788104-GG, N=4 donors; rs12788104-AA, N=4 donors), no sample size calculation was performed. For quantification of MUC5AC staining, in blinded fashion, we took images of 20x objective fields (8-11 fields per condition) and counted the number of MUC5AC positive cells. This sample size was sufficient to identify changes in MUC5AC+ cell counts.

Data exclusions	For GALA II eQTL study, we excluded 14 donors that failed genotyping QC from TOPMED.
Replication	For GALA II eQTL study, no replication was performed, but results are robust due to the large sample size. There are 681 donors with paired genotype and expression data which is larger than the sample size of any single tissue in GTEx.<br><br>For the analysis of MUC5AC ELISA assay data, 6 replicates were performed per donor/condition combination.<br><br>For the MUC5AC immunofluorescence study, we took images of 20x objective fields (8-11 fields per condition).
Randomization	Randomization was not applicable to eQTL analysis as there were no experimental groups. The eQTL analysis was adjusted age, sex, bmi, asthma status, and admixtures estimates to account for confounders.<br><br>For the analysis of FOXA3 RNA-seq expression data, we performed stratified sampling by randomly select five GALA II donors with rs8103278-GG and five GALA II donors with rs8103278-AA.<br><br>For the analysis of MUC5AC ELISA assay data, we performed stratified sampling by randomly select five GALA II donors with rs12788104-GG and five GALA II donors with rs12788104-AA.<br><br>For the MUC5AC immunofluorescence study, we performed stratified sampling by randomly select four GALA II donors with rs12788104-GG and four GALA II donors with rs12788104-AA.
Blinding	Our study results did not involve the combination of subjective decisions or a priori hypotheses, where classical blinding might be needed. Rather we conducted computational analyses and genome-wide statistical tests, then reported the results of these agnostic analyses. Therefore blinding was unnecessary.

Reporting for specific materials, systems and methods

We require information from authors about some types of materials, experimental systems and methods used in many studies. Here, indicate whether each material, system or method listed is relevant to your study. If you are not sure if a list item applies to your research, read the appropriate section before selecting a response.

Materials & experimental systems

n/a	Involved in the study
☐	☒ Antibodies
☐	☒ Eukaryotic cell lines
☒	☐ Palaeontology and archaeology
☒	☐ Animals and other organisms
☐	☒ Human research participants
☒	☐ Clinical data
☒	☐ Dual use research of concern

Methods

n/a	Involved in the study
☒	☐ ChIP-seq
☒	☐ Flow cytometry
☒	☐ MRI-based neuroimaging

Antibodies

Antibodies used	MUC5AC Monoclonal Antibody; ThermoFisher Scientific Catalog #MA1-21907; Clone 45M1; Host/Isotype: Mouse/IgG1<br>AlexaFluor 594 goat anti-mouse IgG (H+L); Invitrogen Catalog #A11005
Validation	ThermoFisher MUC5AC antibody: RRID - AB_560214; antibody has passed Advanced Verifications by Thermo - "The Advanced Verification badge is applied to products that have passed application and specificity testing. This badge can be found in the search results and at the top of the product specific webpages. Data supporting the Advanced Verification badges can be found in product specific data galleries. Product validation and references for Invitrogen secondary antibody can be found here: https://www.thermofisher.com/order/genome-database/dataSheetPdf?producttype=antibody&productsubtype=antibody_secondary&productId=A-11005&version=165

Eukaryotic cell lines

Policy information about cell lines

Cell line source(s)	NIH 3T3 Fibroblast cells were purchased from ATCC for use as feeder co-culture for primary human epithelial cells.
Authentication	No authentication was performed.
Mycoplasma contamination	No mycoplasma testing was performed.
Commonly misidentified lines (See ICLAC register)	No commonly misidentified lines were used.

Human research participants

Policy information about studies involving human research participants

Population characteristics	The Genes-Environment & Admixture in Latino Americans study (GALA II) is an on-going case-control study of asthma in Latino children and adolescents. The study includes subjects with asthma and healthy controls of Latino descent between the ages of 8 and 21.
Recruitment	GALA II Study subjects were recruited from the community centers and clinics in the mainland U.S. and Puerto Rico (2006-present). Asthma case status was physician-diagnosed. Recruited subjects completed in-person questionnaires detailing medical, environmental, and demographic information
Ethics oversight	GALA: University of California San Francisco (UCSF) IRB number 10–00889, Reference number 153543, NJH HS-2627

Note that full information on the approval of the study protocol must also be provided in the manuscript.
